# Case Report: Inflammation and Endothelial Injury Profiling of COVID-19 Pediatric Multisystem Inflammatory Syndrome (MIS-C)

**DOI:** 10.3389/fped.2021.597926

**Published:** 2021-04-08

**Authors:** Douglas D. Fraser, Eric K. Patterson, Mark Daley, Gediminas Cepinskas

**Affiliations:** ^1^Lawson Health Research Institute, London, ON, Canada; ^2^Pediatrics, Western University, London, ON, Canada; ^3^Computer Science, Western University, London, ON, Canada; ^4^Medical Biophysics, Western University, London, ON, Canada

**Keywords:** COVID-19, inflammation, endothelial injury, children, multisystem, MIS-C

## Abstract

**Introduction:** COVID-19 is associated with a novel multi-system inflammatory syndrome that shares some characteristics with Kawasaki's Disease. The syndrome manifestation is delayed relative to COVID-19 onset, with a spectrum of clinical severity. Clinical signs may include persistent fever, gastrointestinal symptoms, cardiac inflammation and/or shock.

**Case Presentation:** We measured 59 inflammatory and endothelial injury plasma analytes in an adolescent girl that presented with malaise, fever, cough, strawberry tongue and jaundice. Her COVID-19 status was positive with detection of 2 SARS-CoV-2 viral genes using polymerase chain reaction. She was treated with intravenous immunoglobulin prior to blood draw, but our plasma measurements suggested a unique analyte expression pattern associated with inflammation, endothelial injury and microvascular glycocalyx degradation.

**Conclusions:** COVID-19 is associated with a multi-system inflammatory syndrome and a unique inflammatory and endothelial injury signature.

**Summary:** Analyte markers of inflammation and endothelial cell injury might serve as putative biomarkers and/or be investigated further as potential therapeutic targets.

## Introduction

COVID-19 has been associated with a novel multisystem inflammatory syndrome in children (1, 2). Presentation is typically delayed after SARS-CoV-2 exposure, with clinical symptoms that share features of Kawasaki's Disease (KD) (3). A “cytokine storm” has been suggested to underlie the syndrome, with tissue injury secondary to the host innate response (4). The inflammatory and endothelial injury mediators have been described for adult COVID-19 patients (5–8), but knowledge of these analytes in COVID-19 children is limited and may be critically important for earlier syndrome recognition and for potential interventions. Furthermore, inflammatory analyte pediatric reference standards for healthy control comparisons are generally unavailable.

## Patient Information

A 15-year-old female presented to hospital to a tertiary care emergency department with a history of malaise, dry cough, strawberry tongue, rash and jaundice. COVID19 was confirmed by detection of two SARS-CoV-2 viral genes using polymerase chain reaction. Her complete blood count, electrolytes, coagulation profile and blood gas were normal. C-reactive protein and ferritin were mildly elevated at 25.7 mg/L and 302 μg/L, respectively. She had a mild hepatitis with alanine aminotransferase 142 U/L, aspartate aminotransferase 87 U/L, alkaline phosphate 405 U/L, total bilirubin 92.6 μmol/L. She was admitted to hospital with a presumptive diagnosis of atypical KD and treated with intravenous immunoglobulin (IVIg) and Aspirin. Her inpatient electrocardiogram and echocardiogram were normal.

Blood was drawn for inflammation/endothelial injury profiling after the patient's COVID-19 status was confirmed, but IVIg had already been administered approximately 48 h earlier. Thus, analyte measurements must be evaluated in the context of this immune modulator (see below). Nonetheless, we measured 59 inflammation- and endothelium-related analytes using multiplexed biomarker immunoassay kits or enzyme-linked immunosorbent assay (ELISA). As only one COVID-19 pediatric patient was admitted to our hospital, we compared the measured analyte values from this COVID-19 case patient to analyte reference ranges that we obtained from a cohort of 20 pediatric healthy control subjects [median 15 years of age (IQR 8)].

**Figure 1:**
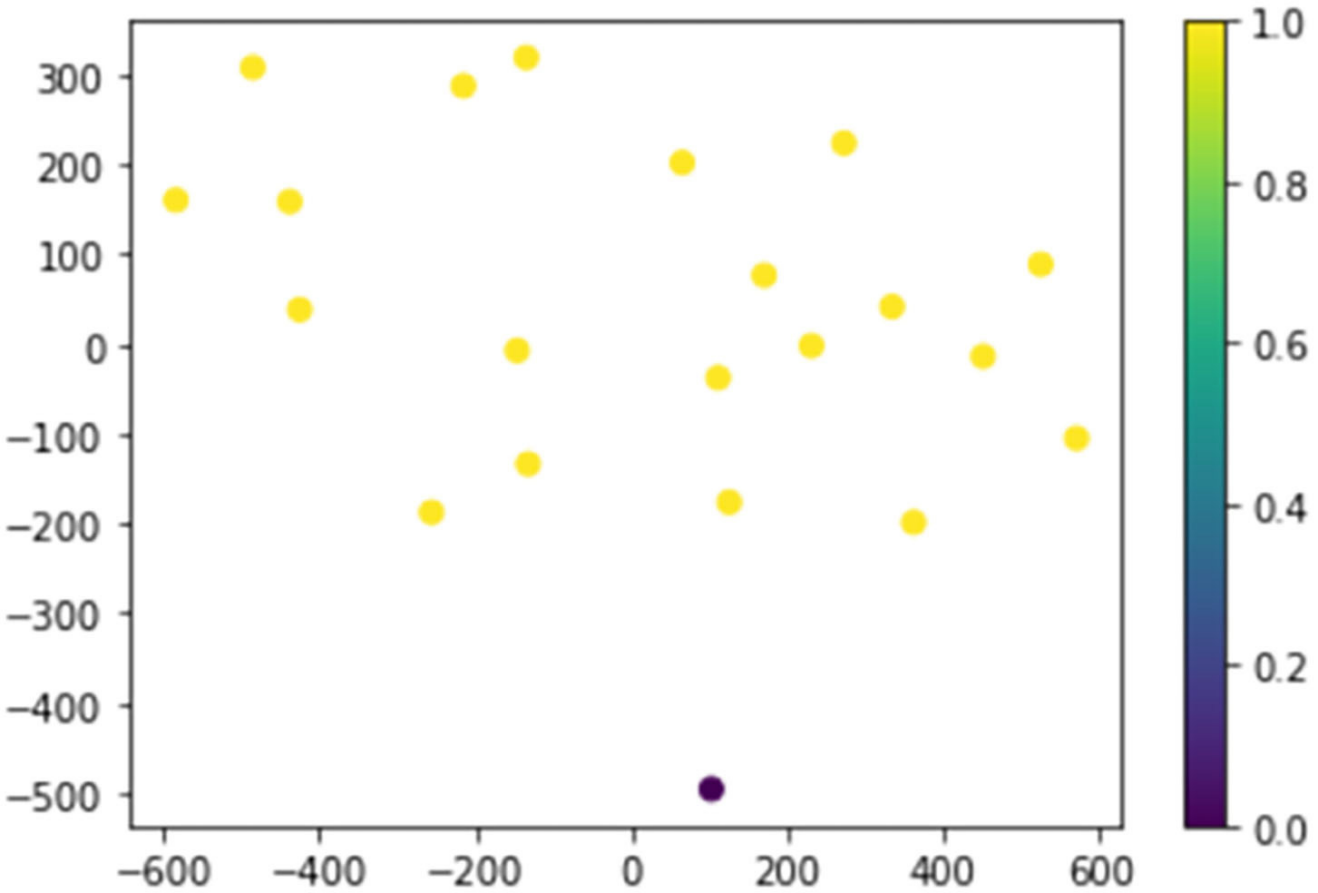
The COVID-19 case patient and 20 healthy control subjects plotted in two dimensions following dimensionality reduction by stochastic neighbor embedding. The Purple dot represents the COVID-19 patient, while the yellow dots represent the healthy controls. The dimensionality reduction shows that based on 59 plasma analyte concentrations, the COVID-19 patients is distinct and easily separable. The axes are dimension-less.

The analyte data from the COVID-19 patient and the 20 healthy control subjects were first visualized with a nonlinear dimensionality reduction on the full data matrix using the t-distributed stochastic nearest neighbor (t-SNE) embedding algorithm (Figure 1) (9). t-SNE assumes that the “optimal” representation of the data lies on a manifold with complex geometry, but low dimension, embedded in the full dimensional space of the raw data. Based on analyte measurements, the COVID-19 case patient is a clear outlier with respect to her inflammation and endothelial injury profile.

We then generated confidence intervals (CIs) for the expected value of each analyte using the plasma measurements from the 20 healthy pediatric controls. The plasma values for each analyte were not normally distributed, so we computed 99.9% (95%, Bonferonni corrected for comparison across 59 plasma analytes) CIs via the bias corrected and accelerated bootstrap. Plasma analyte values in the COVID-19 case patient that were outside the CIs for healthy control subjects were therefore considered significant (*p* < 0.05, corrected; Table 1). We found significant elevations in 21 inflammation and endothelial analyte markers, while 1 endothelial glycocalyx degradation marker (heparan sulfate) was significantly depressed (Table 1).

**Table 1:** Significant analyte concentration changes for the COVID-19 case patient and 20 healthy control subjects (EC, endothelial cell).

**Plasma analyte**	**Units**	**20 healthy controls (CI: 5%, 95%)**	**Case patient**
1. MMP7	pg/ml	3,356, 4,424	51,788
2. IP-10	pg/ml	86, 242	1,098
3. Resistin	pg/ml	7.3, 11.1	41.8
4. IL-3	pg/ml	0.1, 2.4	7.3
5. Hyaluronic acid (EC)	ng/ml	17.6, 40.4	119.2
6. Thrombospondin-1	pg/ml	620, 1,275	3,286
7. Elastase 2	pg/ml	2.1, 4.4	11.3
8. PDGF-AB/BB	pg/ml	769, 2,537	6,390
9. MIG	pg/ml	1,205, 2,684	6,531
10. MCP-1	pg/ml	190.3, 269.5	529.4
11. MMP1	pg/ml	384, 709	1,286
12. Lactoferrin	pg/ml	338.3, 521.3	845.8
13. IL-1RA	pg/ml	8.1, 73.3	112.0
14. IL-18	pg/ml	30.3, 64.6	98.3
15. IFNα2	pg/ml	13.2, 120.7	179.6
16. sP-selectin (EC)	ng/ml	16.3, 22.4	30.4
17. MIP-1β	pg/ml	22.8, 72.0	89.16
18. Eotaxin	pg/ml	49.4, 81.6	97.4
19. MMP8	pg/ml	288.4, 643.6	762.7
20. PDGF-AA	pg/ml	84.4, 652.9	766.8
21. MMP10	pg/ml	385.2, 751.4	876.1
22. Heparan sulfate (EC)	ng/ml	22.7, 294.5	20.6

*The data is in rank order of magnitude of change*.

After 3 days of observation, and partial resolution of her symptoms, our COVID-19 case patient was discharged home on Aspirin (3 mg/kg/day). Follow-up appointments at 2- and 6-weeks post-discharge revealed resolving hepatitis and both electrocardiograms and echocardiograms were normal with no evidence of coronary aneurysm. The aspirin was discontinued at the 6-week follow-up appointment.

## Discussion

We present an COVID-19 positive adolescent female with a unique analyte expression pattern associated with inflammation, endothelial injury and microvascular glycocalyx degradation. Given the timing of the blood draw, some plasma analytes may have been altered by IVIg administration or down-regulated to the levels of healthy control subjects. Nonetheless, we briefly summarize the purported actions of the top five analytes that were elevated 3-fold or greater in the COVID-19 case patients relative to the upper confidence limit for healthy controls, and we compare these findings to published reports of IVIg-treated KD patients (10–13).

Matrix metalloproteinase 7 (MMP7) was the most elevated analyte in the COVID-19 case patient relative to healthy control subjects. Also called matrilysin, MMP7 is expressed in endothelial cells, monocytes and macrophages and it is capable of degrading multiple extracellular membrane components (proteoglycans, laminin, fibronectin, casein and basement membrane collagen type IV). MMP7 is significantly upregulated in KD and it is implicated in acute vasculopathy (14). Specifically, MMP7 degrades endothelial junctions, which can promote vascular leak/edema and/or leukocyte migration into tissues (15). A variety of circulating MMPs are elevated in KD, with plasma levels decreasing following IVIg therapy (16).

Interferon-γ-inducible protein 10 (IP-10), an inflammatory cytokine secreted primarily by monocytes and endothelial cells in response to interferon-γ (IFN γ), was also significantly elevated in the COVID-19 case patient. IP-10 has multiple roles including lymphocyte chemoattraction and adhesion to endothelial cells. IP-10 is a promising target for the treatment of infectious diseases as it aids cellular targeting to threatened tissues where it modulates innate and adaptive immune responses. High serum IP-10 is found in KD, and it has been suggested as a KD biomarker (11). IP-10 levels remain elevated after IVIg treatment in some refractory KD patients (10), while in others, IP-10 slowly decreased to control levels by 1-week post-IVIg administration (11).

Resistin is highly expressed in macrophages, bone marrow and the non-fat fraction of adipose tissue, and it stimulates several pro-inflammatory pathways and cytokines. Microvascular tone, as well as endothelial cell barrier function and nitric oxide production, are all altered by resistin. Similar to our COVID-19 case patient, elevated resistin is found in plasma from KD patients (17, 18). Post-IVIg therapy, 3–5 days, plasma resistin levels in KD patients significantly decreased (12).

Interleukin 3 (IL-3), released by activated T-cells, was elevated in our COVID-19 case patient. IL-3 promotes the production of inflammatory monocytes and neutrophils, thereby contributing to the cytokine storm that is implicated in sepsis from multiple etiologies. The microvascular endothelial cell response to inflammation and immunity is also regulated by IL-3 (19) and vasculopathy is suggested to be a primary feature of the novel multi-system inflammatory syndrome. IVIg treatment decreases IL-3 production by activated T-cells and monocyte/macrophage to control levels within 72 h (20).

Hyaluronic acid is a major constituent of the microvascular glycocalyx, an extracellular matrix that coats the luminal surface of the endothelium (21). Hyaluronic acid degradation products are significantly elevated in plasma from the COVID-19 case patient, suggesting that the microvascular endothelial cell luminal surface has been pathologically altered. Disruption of the endothelial glycocalyx is associated with vascular lesions and barrier dysfunction in KD (13), as well as decreased endothelial nitric oxide production and increased platelet/endothelium adhesion (21), Endothelial cell injury was supported in the COVID-19 case patient by the parallel elevation of soluble P-selectin, an endothelial/platelet glycoprotein that mediates adhesive intercellular interactions (22). Elevated plasma hyaluronic acid found in KD patients slowly decreases after IVIg treatment, normalizing at the time of discharge in the convalescent phase (13).

Our measurements showed minimal alterations in 37 inflammation and endothelial analyte markers: epidermal growth factor (EGF), granulocyte-colony stimulating factor (G-CSF), granulocyte-macrophage colony-stimulating factor (GM-CSF), IFNγ, interleukin 1a (IL-1a), IL-1b, IL-2, IL-4, IL-5, IL-6, IL-7, IL-8, IL-10, IL-12(p40), IL-12(p70), IL-13, IL-15, IL-17a, IL17e/IL25, IL-17f, IL-22, macrophage colony-stimulating factor (M-CSF), macrophage inflammatory protein 1α (MIP-1α, tumor necrosis factor α (TNFα), TNFβ, vascular endothelial growth factor A (VEGFA), regulated upon activation, normal T Cell expressed and presumably secreted (RANTES), MMP2, MMP3, MMP9, MMP12, MMP13, neutrophil gelatinase-associated lipocalin (NGAL), Granzyme B, heat shock protein 70 (HSP-70), chondroitin sulfate and syndecan-1. As some of these measurements may have been depressed by IVIg administration (23), no significant conclusions can be made with regards to their pre-treatment level. It is also plausible that some inflammatory analytes were transiently increased with inflammation onset, with TNF and IL-6 as typical examples (24). TNF-α is a pro-inflammatory cytokine released primarily by monocytes and macrophages that enhances the adaptive immune response. IL-6 is produced by monocytes and macrophages, and induces T-cell activation, B cell proliferation and stimulates the acute phase reaction, all of which lead to augmentation of the immune response.

## Conclusion

Pediatric COVID-19 patients can present with a novel multisystem inflammatory syndrome with some clinical features similar to KD. The analyte measurements presented in this study, albeit post IVIg treatment, support a systemic inflammatory process that resulted in significant endothelial injury. The elevated analyte measurements are similar to KD patients whom had also received IVIg therapy. These data should aid future hypothesis-generating research, as some of the identified analytes might be putative disease biomarkers and/or potential therapeutic targets. Age-matched healthy control analyte reference values are included and will be valuable for future studies.

## Data Availability Statement

The original contributions generated for the study are included in the article/supplementary material, further inquiries can be directed to the corresponding author/s.

## Ethics Statement

The studies involving human participants were reviewed and approved by Human Ethics Board, Western University. Written informed consent to participate in this study was provided by the participants' legal guardian/next of kin.

## Author Contributions

DF conceived the study, obtained ethic approval and consent, collected the sample, and measured the analytes and wrote the manuscript. EP measure analytes and revised the manuscript. MD analyzed the analyte measurements and revised the manuscript. GC measured the analytes and revised the manuscript. All authors contributed to the article and approved the submitted version.

## Conflict of Interest

The authors declare that the research was conducted in the absence of any commercial or financial relationships that could be construed as a potential conflict of interest.
